# Extreme microbes for a changing planet and beyond

**DOI:** 10.1093/sumbio/qvag041

**Published:** 2026-10-05

**Authors:** Alexandre Soares Rosado

**Affiliations:** Biological and Environmental Science and Engineering Division, King Abdullah University of Science and Technology, Thuwal 23955-6900, Saudi Arabia

**Keywords:** extremophiles, microbial biotechnology, sustainability, climate adaptation, microbial ecology, space biology

Our planet faces increasingly extreme conditions as environmental change accelerates. However, extremes are common in nature, and what we consider extreme environments often reflect human expectations rather than biological limits. Frozen Antarctic soils, hypersaline mangroves, volcanic craters, and deep-sea hydrothermal vents may appear hostile, but microorganisms thrive, interact, and sustain essential biological processes in all of these settings. They are not merely surviving at the margins of life; instead, they demonstrate how life persists and adapts under conditions that exceed the tolerance of most organisms.

This matters because such conditions are no longer limited to remote areas. Heatwaves, droughts, soil salinization, wildfires, floods, pollution, and melting glaciers are now transforming landscapes worldwide. Conditions once considered rare are increasingly common in agricultural regions, coastal ecosystems, and populated areas. Microbial lineages have faced many of these stresses over evolutionary timescales and evolved diverse strategies to survive them. The key question is whether we can learn from those strategies quickly enough. As environmental extremes are normalized, research on extremophiles and other stress-tolerant microorganisms must become central to sustainability efforts.

Extremophiles have long contributed to modern biotechnology. The most well-known example is Taq polymerase, derived from the thermophilic bacterium *Thermus aquaticus*. Its introduction into the polymerase chain reaction revolutionized DNA amplification, making repeated cycles practical and automatable. This development markedly impacted molecular biology, diagnostics, and medicine (Saiki et al. 1988). Taq’s importance extends beyond the enzyme itself; it exemplifies how a molecular adaptation for high-temperature survival became the basis of a major technological advance.

Thermophiles, halophiles, psychrophiles, acidophiles, and other stress-adapted microorganisms have enzymes, protective compounds, and metabolic pathways that operate beyond the limits of many standard biological systems. Conditions such as high temperature, salinity, extreme pH, or low water availability can be advantageous in industrial processes rather than obstructive. When properly managed, these microorganisms can reduce contamination risk, streamline product recovery, or enable the use of saline waste, lignocellulosic residues, and other industrial raw materials that conventional enzymes process inefficiently (Cowan et al. 2024, Zheng et al. 2025).

My perspective has been influenced by work across diverse ecosystems, ranging from Antarctica to hypersaline mangroves, deserts, volcanic sites, and deep-sea hydrothermal systems. I find it notable that, despite their apparent differences, the organisms inhabiting these environments often share related biological strategies. For example, microorganisms survive environmental stresses through metabolic adaptability, protective pigments, efficient nutrient uptake, DNA repair, dormancy, and interactions with other organisms. Although certain environments appear distinct, their microorganisms frequently use a similar biological “language.”

Antarctica highlights the importance of ecological context in adaptation. For instance, addressing petroleum contamination cannot rely solely on protocols developed for temperate environments. Low temperatures and scarce nutrients constrain microbial degradation, and the sensitive polar ecosystems are easily disrupted by poorly suited interventions. Research on an old diesel spill on the Antarctic Peninsula demonstrated that effective remediation must consider the native microbial communities’ physiological capacities and the environmental conditions that influence them (de Jesus et al. 2015).

The same environment can yield functions with value beyond their original ecological context. For example, carotenoid extracts from an ultraviolet (UV)-resistant *Arthrobacter* strain, isolated on King George Island, showed UV-B photoprotection in experimental assays (Paredes Contreras et al. 2025). The Antarctic examples therefore illustrate two routes from environmental adaptation to application. Locally adapted communities may be used where they already function under specific site conditions, whereas individual compounds or traits can be isolated and tested in other settings.

Agriculture provides both opportunities. Plant-associated microorganisms can mobilize nutrients, alter hormone signaling, modify root growth, and support plants in tolerating water or stress. For example, a defined microbial community assembled from the root microbiota of the desert shrub *Caragana korshinskii* has been shown to enhance drought tolerance in wheat (Hao et al. 2026). These findings shift the focus from simply identifying stress-adapted microorganisms to experimentally demonstrating their effects on plant performance under water limitation.

Field conditions are more complex than typical laboratory tests. Drought often coincides with heat and high salinity, pollution with nutrient limitation, and radiation with desiccation and oxidative stress. A strain selected for a specific trait under a single controlled condition may lose effectiveness when multiple stresses occur simultaneously. Therefore, candidate organisms and communities should be tested under stress combinations that mimic their expected environmental conditions (Chasapi et al. 2026).

Accordingly, relying solely on a single isolate may not be an effective approach. Organisms linked by metabolic exchange and division of labor often collectively perform environmental functions, including nutrient cycling, soil recovery, and contaminant transformation. Defined microbial communities can combine complementary functions while retaining known composition and experimental reproducibility (Koch et al. 2026). However, their effectiveness depends on factors such as compatibility, stability, establishment in the recipient environment, and actual performance, all of which must be verified.

Hydrothermal systems underscore this community-level organization. Genome-resolved analyses of Red Sea hydrothermal environments have revealed high microbial diversity and potential interactions involving carbon, nitrogen, sulfur, iron, and other elemental cycles (Altalhi et al. 2025). These environments are valuable not only for hosting unique species but also for examining how microbial communities sustain their functions under several concurrent constraints. Their scientific and biotechnological value further supports the need for protection. Extreme habitats are ecosystems, not just reservoirs of genes and organisms. Destructive exploitation may eliminate biological resources before they are fully understood (Cowan et al. 2024).

Plastic pollution presents a related translational challenge. Conventional recycling methods are limited by technical, economic, and environmental factors, highlighting the need for complementary biological solutions. In collaborative research on saline mangrove soils, our group and partners identified candidate polyethylene terephthalate–degrading enzymes from halophilic microbial communities enriched on lignocellulosic material (Peña-Valencia et al. 2026). Their activity and industrial application still require experimental validation. Nonetheless, this research demonstrates how ecological selection, community analysis, and functional screening can identify promising biocatalysts that may otherwise go undetected.

Metagenomics, integrated multiomics, bioinformatics, and artificial intelligence are accelerating this process by facilitating identification of adaptive traits, inference of interactions, and selection of uncharacterized proteins for testing (Chasapi et al. 2026).

Much of extremophile biotechnology still follows a discovery-first model. Researchers identify and characterize an organism, gene, or enzyme before seeking practical uses. A complementary approach would start with a clearly defined industrial or environmental need and then search for biological functions that meet those requirements. This demand-led model would not replace fundamental discovery but could shorten the path from interesting biology to useful technology (Cowan et al. 2024).

Prediction, however, is only one stage of development. A candidate gene, strain, or community must show reproducible function, remain stable under realistic operating conditions, and outperform existing options. Factors such as cultivation, formulation, biosafety, regulation, production costs, and environmental impact then determine whether laboratory results can translate into practical technology. Industry and regulatory partners should be involved early enough to influence these requirements, rather than being approached only after the biological system has been optimized in isolation.

Space exploration offers a highly challenging environment in which to test progression from adaptation to application. Human missions to the Moon or Mars will require reliable biological systems to produce food, recycle water, manage waste, recover nutrients, and extract useful materials from local sources. Engineering solutions alone cannot fulfill all these needs. Sustained human presence beyond Earth will depend on biological processes, with microorganisms expected to be pivotal (Lopez et al. 2019).

Food production provides a clear example. During NASA-supported research in Yellowstone, the acid-tolerant fungus *Fusarium* sp. strain flavolapis was isolated from a geothermal environment and later used as the basis for the microbial protein Fy. This organism was then cultivated aboard the International Space Station to examine its potential as a food-production platform for long missions (NASA 2022). Its use in terrestrial food production and space experiments demonstrates how the same microorganism can be investigated under vastly different resource constraints.

Additional microbial functions are being explored under space-relevant conditions. Experiments on the International Space Station showed that microbes can extract rare earth elements under microgravity and simulated Martian gravity (Cockell et al. 2020). Laboratory tests revealed that the melanized fungus *Rhinocladiella similis* can survive combined UV-C radiation and perchlorate exposure under conditions mimicking Martian stressors (dos Santos et al. 2025). These findings do not justify releasing terrestrial microbes on Mars; instead, they demonstrate that microbial physiology can be tested under extraterrestrial conditions and assessed for specific functions.

I view space exploration as an extreme test of sustainability. Habitats on the Moon or Mars cannot rely indefinitely on resource extraction, waste generation, and waiting for supplies from Earth. Water must be recycled, nutrients recovered, and waste repurposed as resources. Biological systems must operate with limited energy and minimal external inputs. The technologies developed under these constraints could also improve agriculture, water recycling, waste management, and resource recovery on Earth, especially in challenging environments. Therefore, space research serves as a stringent test of sustainability, rather than as a separate technological pursuit.

Any use of microorganisms beyond Earth must align with planetary protection principles. Research on extremophile microbiomes and Mars has emphasized the importance of considering ecological interactions, potential impacts on extraterrestrial environments, safeguarding biosignatures, and ensuring responsible governance (Coleine et al. 2025). Traits that make microorganisms valuable for space applications also justify the need to carefully manage their use and deployment.

Our goal is not simply to discover organisms that tolerate extreme conditions but to identify which functions remain beneficial after experimental validation, environmental testing, and practical assessment. Achieving this will require collaboration across microbial ecology, computational biology, agriculture, engineering, industry, environmental science, and space research. No single laboratory can address a planetary challenge.

Extremophiles will not resolve the environmental crisis, but they can deepen our understanding of life’s possibilities under resource scarcity and harsh conditions. To better comprehend our planet’s future, we should study organisms that thrive in extreme environments. As climate change shifts ecosystems toward unfamiliar conditions and human exploration extends beyond Earth, the smallest life forms could become vital allies in building a more resilient future.

## Data Availability

No data were used for the research described in this article.
